# Increased Serum Hepcidin Levels in Subjects with the Metabolic Syndrome: A Population Study

**DOI:** 10.1371/journal.pone.0048250

**Published:** 2012-10-29

**Authors:** Nicola Martinelli, Michela Traglia, Natascia Campostrini, Ginevra Biino, Michela Corbella, Cinzia Sala, Fabiana Busti, Corrado Masciullo, Daniele Manna, Sara Previtali, Annalisa Castagna, Giorgio Pistis, Oliviero Olivieri, Daniela Toniolo, Clara Camaschella, Domenico Girelli

**Affiliations:** 1 Department of Medicine, University of Verona, Verona, Italy; 2 Division of Genetics and Cell Biology, San Raffaele Research Institute, Milan, Italy; 3 Vita Salute San Raffaele University, Milan, Italy; Universita Magna-Graecia di Catanzaro, Italy

## Abstract

The recent discovery of hepcidin, the key iron regulatory hormone, has changed our view of iron metabolism, which in turn is long known to be linked with insulin resistant states, including type 2 diabetes mellitus and the Metabolic Syndrome (MetS). Serum ferritin levels are often elevated in MetS (Dysmetabolic hyperferritinemia - DHF), and are sometimes associated with a true mild-to-moderate hepatic iron overload (dysmetabolic iron overload syndrome - DIOS). However, the pathophysiological link between iron and MetS remains unclear. This study was aimed to investigate, for the first time, the relationship between MetS and hepcidin at population level. We measured serum hepcidin levels by Mass Spectrometry in 1,391 subjects from the Val Borbera population, and evaluated their relationship with classical MetS features. Hepcidin levels increased significantly and linearly with increasing number of MetS features, paralleling the trend of serum ferritin. In multivariate models adjusted for relevant variables including age, C-Reactive Protein, and the HFE C282Y mutation, ferritin was the only significant independent predictor of hepcidin in males, while in females MetS was also independently associated with hepcidin. Overall, these data indicate that the fundamental iron regulatory feedback is preserved in MetS, i.e. that hepcidin tends to progressively increase in response to the increase of iron stores. Due to recently discovered pleiotropic effects of hepcidin, this may worsen insulin resistance and contribute to the cardiovascular complications of MetS.

## Introduction

The “metabolic syndrome” (MetS) is a condition highly prevalent in western countries, involving near one fourth of the adult population [1]. Although definitions vary, the essential features of MetS are represented by the deadly quartet of hyperglycemia, dyslipidemia, hypertension, and obesity [2], leading to a substantial cardiovascular risk, but also to risk of hepatic diseases, namely nonalcoholic fatty liver disease (NAFLD). In 1997, Moirand et al. first reported the presence of histologically proven liver iron overload in overweight subjects with abnormal glucose metabolism and dyslipidemia [3]. This condition, later designated as dysmetabolic iron overload syndrome (DIOS) [4], is now known to occur in about one third of subjects with NAFLD and represents the most severe counterpart of the so-called dysmetabolic hyperferritinemia (DHF) (for a recent extensive review, see Dongiovanni et al [5]). The latter in turn is by far the commonest cause of consultation for increased serum ferritin levels in clinical practice [6]. Nevertheless, the complex pathophysiological links between iron and metabolic derangements remain poorly understood [5]. In the last ten years, hepcidin has emerged as the key iron-regulatory hormone [7]. This defensin-like 25 amino acid peptide is mainly produced by the liver in response to increased plasma or tissue iron to homeostatically down-regulate absorption and recycling of the metal [8]. At the molecular level, hepcidin acts by binding and inactivating its cell membrane receptor ferroportin, the only known cellular iron exporter [9]. Ferroportin is particularly expressed by cells critical for iron homeostasis, like absorbing duodenal enterocytes, reticuloendothelial macrophages (involved in iron storage and recycling), and hepatocytes (involved in iron storage and endocrine regulation) [9]. Hepcidin is also upregulated by inflammatory cytokines, a response believed to contribute to host defense by subtracting iron from invading pathogens [10]. Given its central role in iron homeostasis, hepcidin represents an appealing candidate to be investigated in subjects with MetS features, but until now methodological difficulties [11] have hampered large epidemiological studies. Taking advantage from the recently completed iron section of the Val Borbera Study (VBS) [12], this study was aimed to investigate the relationships between hepcidin and the main features of MetS at population level.

## Materials and Methods

Details on the VBS population have been previously reported elsewhere [12]. Individuals aged 18 years or older were eligible to participate in the study. In this analysis we included subjects with available complete data allowing their classification according to established criteria for MetS [2]. In detail, the following features were considered: 1) abdominal obesity, defined as the presence of waist circumference ≥94 cm in men or ≥80 cm in women; 2) fasting plasma glucose ≥100 mg/dL or drug treatment for elevated blood glucose; 3) serum triglycerides ≥150 mg/dL or drug treatment for elevated triglycerides; 4) serum HDL cholesterol (HDL-C) <40 mg/dL in men and <50 mg/dL in women or drug treatment for low HDL-C; 5) blood pressure ≥130/85 mmHg or drug treatment for elevated blood pressure. Subjects were considered to have MetS when they had at least three of the above-mentioned five traits. Homozygotes for the hemochromatosis mutation (C282Y on the HFE gene) were excluded (n = 7). A total of 1,391 subjects, 616 men and 775 women were finally included in the present study. Fasting blood samples obtained early in the morning were analyzed the same day or stored at −80°C for further analysis. Routine blood parameters and serum hepcidin were determined by standard methods and by mass spectrometry, respectively, as previously described [12]. The study was approved by the ethical committees of San Raffaele Hospital Milano, Regione Piemonte, and Azienda Integrata Ospedaliera Universitaria of Verona, Italy. All subjects gave written informed consent.

### Statistical Analyses

All calculations were performed using SPSS 17.0 software (SPSS Inc., Chicago, IL, USA). As many of the continuous variables of interest, including serum hepcidin and ferritin, showed a non-Gaussian distribution, their values were log-transformed and expressed as geometric means with 95% confidence intervals (CIs).

Since some subjects had serum hepcidin levels below the lower limit of detection (LLOD) for our method (0.55 nM), to allow a correct analysis these subjects were considered as having hepcidin “0”, and hepcidin was log-transformed after the addition of 0.1 to each value in the dataset. Quantitative data were analyzed using the Student’s t test or by analysis of variance (ANOVA) with polynomial contrasts for linear trend, when appropriate. Qualitative data were analyzed with the χ^2^ test and with χ^2^ analysis for linear trend, when appropriate. Correlations between quantitative variables were assessed using Pearson’s coefficient. Most of the hepcidin-related analyses were done separately in males and females, since we [12] and others [13] recently reported substantial gender differences in hepcidin serum levels. Particularly, women during the fertile period showed hepcidin levels significantly lower (i.e. less than half) than men of the same age range. Similarly, since MetS subjects were older than those without MetS, analyses were always adjusted for age. Independent determinants of serum hepcidin levels were assessed through a series of linear regression models, using either MetS by itself or individual MetS features as covariates, and adjusting for age, ferritin, C-Reactive Protein (CRP) and C282Y HFE mutation. Two-sided p values <0.05 were considered statistically significant.

## Results

**Figure 1:**
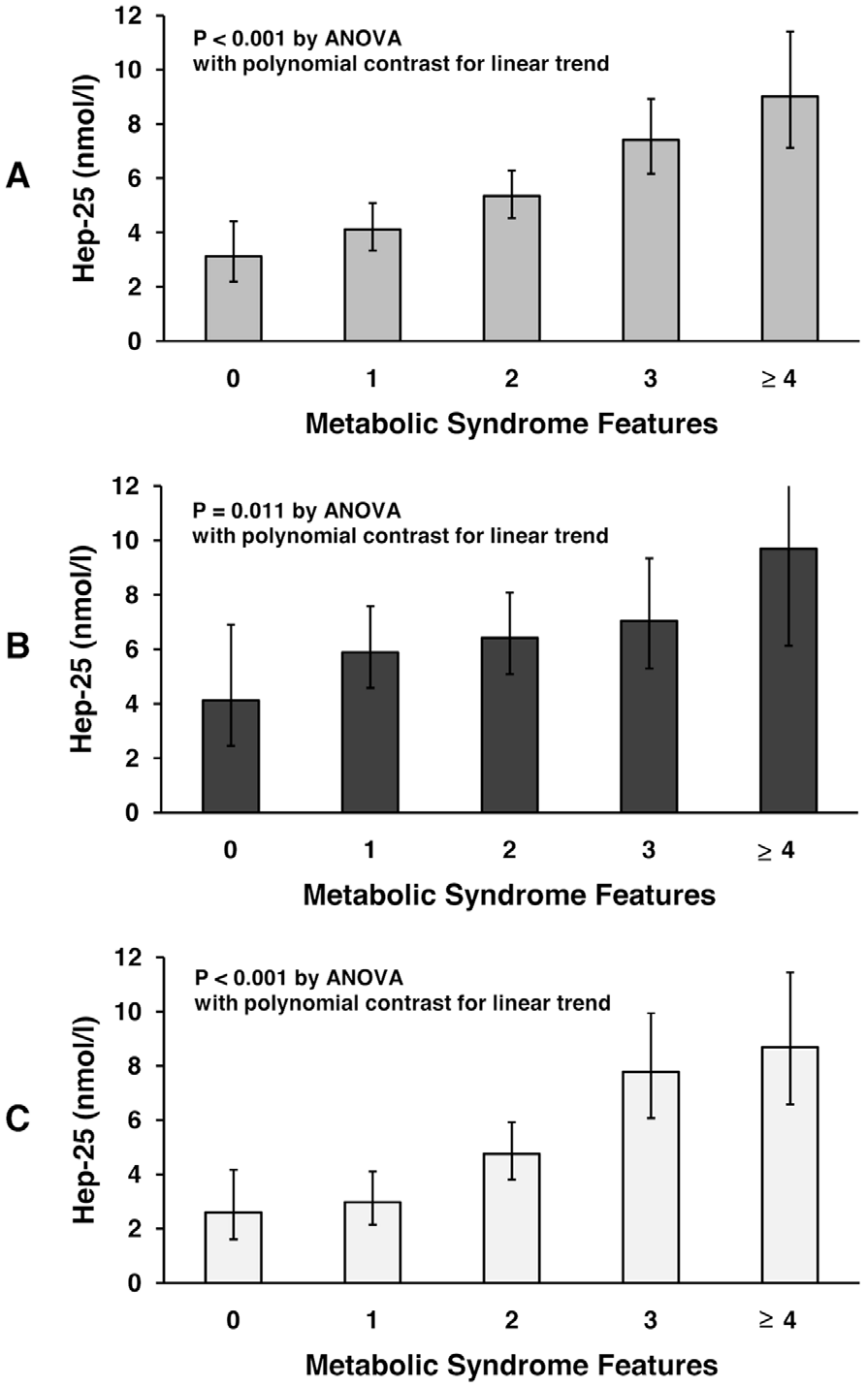
Serum hepcidin levels in the Val Borbera population according to increasing number of MetS features. (A) whole population, (B) males and (C) females.

Table 1 summarizes the main clinical, anthropometrical and biochemical features of the population studied, including stratification by gender. Using these data, we calculated the population prevalence of MetS features (shown in Table S1). Overall, 304 individuals (21.9%) could be classified as having MetS using the criteria defined above. Table 2 shows the biochemical iron parameters of the VB subjects stratified for having or not the MetS. Of note, MetS subjects had significantly higher serum levels of both ferritin and hepcidin as compared to subjects without MetS (geometric means for ferritin: 102 versus 61 µg/L, P<0.001; for hepcidin: 7.95 versus 4.29 nM, P<0.001). Such results remained statistically significant also after adjusting for age and sex (Table 2, last column). Beyond mean values, to evaluate the proportion of MetS individuals with high hepcidin values, we stratified hepcidin levels into quartiles considering subjects with no or just one MetS feature as the reference group. Of note, subjects with undetectable hepcidin levels were significantly underrepresented in the MetS group as compared to the non-MetS group (Table 2). As shown in Figure S1, near 50% of subjects with ≥4 MetS features had hepcidin values in the top quartile of the reference group. We then evaluated the behavior of these two parameters according to the number of MetS features (0 to 5, where the last two categories were merged because of the small number of subjects with all five the features). According to our previous data in a different population [14], serum ferritin levels increased linearly according to increasing number of MetS features, both in males and in females (Figures S2 A–C). The same behavior was observed for serum hepcidin levels, again both in males and in females (Figures 1 A–C). At univariate analyses (Table S2), the variable showing the strongest association with hepcidin was ferritin (beta coefficients  = 0.559, and 0.585 in males and females, respectively; P<0.001 for both). Of note, beta coefficients and slopes were quite similar when correlations were made separately in subjects with or without the MetS (Figure S3), suggesting that the homeostatic loop of hepcidin in response to iron stores is well preserved in MetS.

**Table 1:** Clinical, anthropometrical, and biochemical data of the VB population.

	All (n = 1,391)	Male (n = 616 )	Female (n = 775)	*P*
**Age (years)**	55.8±18.2	55.6±17.8	56.0±18.4	0.715
**Weight (Kg)**	70.0±14.1	77.8±12.3	63.7±12.3	<0.001
**Waist (cm)**	90.0±11.8	92.5±10.3	88.0±12.5	<0.001
**Height (cm)**	164.1±11.4	171.7±7.3	158.0±10.5	<0.001
**BMI**	25.9±4.5	26.4±3.8	25.4±4.9	<0.001
**Total Cholesterol (mg/dl)**	203±42	199±41	207±42	<0.001
**HDL Cholesterol (mg/dl)**	59±15	54±13	63±15	<0.001
**LDL Cholesterol (mg/dl)**	124±35	122±34	125±36	0.122
**Triglycerides** * **(mg/dl)**	89 (87–92)	98 (94–102)	83 (80–86)	<0.001
**Glucose** * **(mg/dl)**	90 (89–91)	92 (91–94)	88 (87–89)	<0.001
**Diabetes %**	3.0	2.6	3.3	0.481
**Uric acid (mg/dl)**	5.0±1.3	5.8±1.2	4.5±1.1	<0.001
**Creatinine** * **(mg/dl)**	0.85 (0.84–0.86)	0.97 (0.95–0.98)	0.77 (0.76–0.78)	<0.001
**CRP** * **(mg/l)**	0.17 (0.16–0.18)	0.16 (0.15–0.17)	0.17 (0.16–0.18)	0.207
**s-Iron (µg/l)**	97.3±34.3	105.5±36.5	90.7±30.9	<0.001
**Transferrin (mg/dl)**	241.2±43.5	235.3±38.3	245.9±46.8	<0.001
**Transferrin saturation (%)**	29±11	32±12	27±10	<0.001
**Ferritin** * **(µg/l)**	68 (65–72)	116 (109–124)	45 (42–48)	<0.001
**Hb (g/dl)**	14.4±1.5	15.4±1.2	13.6±1.3	<0.001
**Hematocrit (%)**	43.6±4.1	45.9±3.3	41.8±3.8	<0.001
**RBC (10^6^/µl)**	4.8±0.5	5.1±0.4	4.6±0.4	<0.001
**MCV (fl)**	90.7±6.1	91.1±4.9	90.4±6.9	0.027
**Hepcidin** * **(nmol/l)**	4.9 (4.5–5.3)	6.8 (6.1–7.6)	3.8 (3.4–4.3)	<0.001
**Hepcidin undetectable (n/%)**	152/10.9%	37/6.0%	115/14.8%	<0.001
**Hepcidin/ferritin** * **Ratio (nmol/µg × 1000)**	71.9 (67.3–76.9)	58.8 (53.9–64.0)	84.5 (76.7–93.0)	<0.001

* variables not normally distributed were log-transformed and expressed as geometric means with 95% CIs.

**Table 2:** Biochemical iron parameters in the VB population stratified for presence or absence of MetS.

	Metabolic Syndrome NO(n = 1,087)	Metabolic Syndrome YES(n = 304)	*Unadjusted P*	*Sex- and age-Adjusted P*
**Age (years)**	53.1±18.6	65.6±12.2	<0.001	
**Male Sex (%)**	44.6	43.1	0.636	
**s-Iron (µg/l)**	97.7±34.9	95.6±31.9	0.342	0.721
**Transferrin (mg/dl)**	243.4±44.1	233.5±40.6	<0.001	0.046
**Transferrin saturation (%)**	29±11	30±12	0.510	0.575
**Ferritin** * **(µg/l)**	61 (58–65)	102 (92–112)	<0.001	<0.001
**Hepcidin** * **(nmol/l)**	4.3 (3.9–4.7)	8.0 (7.0–9.1)	<0.001	<0.001
**Hepcidin undetectable (n/%)**	140/12.9%	12/3.9%	<0.001	<0.001
**Hepcidin/Ferritin** * **Ratio (nmol/µg × 1000)**	70.4 (65.2–76.0)	77.8 (68.5–88.3)	0.222	0.093
**Hb (g/dl)**	14.3±1.5	14.6±1.5	0.003	<0.001

* variables not normally distributed were log-transformed and expressed as geometric means with 95% CIs.

We then performed a series of multiple logistic models to assess the influence of MetS or its individual components on hepcidin levels in both sexes after adjustment for age and all the other relevant covariates, i.e. ferritin, CRP, and C282Y HFE mutation (whose allelic frequency in the VB population was 0.065) [12]. When considering MetS as a comprehensive covariate (Table 3) in a model adjusted for age and serum ferritin, it was independently associated with hepcidin in females but not in males, although the standardized beta coefficient (0.093) for MetS was quite lower than that for ferritin (standardized beta coefficient  = 0.580). This association remained statistically significant also after adjustment for CRP and C282Y HFE mutation (standardized beta coefficient  = 0.080; P = 0.012), as well as after including in the model hemoglobin, uric acid, and creatinine (standardized beta coefficient  = 0.073; P = 0.028). Considering the individual MetS features as covariates (Table S3), the only independent association was observed for abnormal glucose metabolism in females, again with a beta coefficient (0.080) much lower than that of ferritin (0.638). Since the interaction term between ferritin and MetS was significant in females (P<0.001), hepcidin levels were stratified in this group according to both ferritin levels and presence/absence of MetS. As shown in Figure S4, the MetS-associated increase of hepcidin was particularly evident (and statistically significant) in females with the lower ferritin values. A similar trend was not observed in males (data not shown). Finally, when females were stratified on the basis of hepcidin levels, the prevalence of MetS increased progressively from the lowest to the highest strata (Figure S5A). This association remained statistically significant after adjustment for age and ferritin (Figure S5B).

**Table 3:** Predictors of hepcidin-25 in males and females, considering MetS as a comprehensive binary (present versus absent) covariate.

	Male		Female	
	β-coefficient	*P*	β-coefficient	*P*
**Age (years)**	−0.056	0.098	−0.050	0.135
**Metabolic Syndrome**	−0.040	0.244	0.093	0.003*
**S-Ferritin**	0.569	<0.001	0.580	<0.001

* this association remained statistically significant also after adjusting for CRP, C282Y HFE mutation, hemoglobin, uric acid, and creatinine.

## Discussion

In the recent years, a bulk of evidence, particularly from epidemiological studies [14]–[17] have established a link between iron metabolism and insulin resistant states, including type 2 diabetes mellitus and the MetS (for recent reviews, see Dongiovanni et al [5] and Rajpathak et al [18]). Accordingly, experimental studies [19], recently confirmed by a sophisticated approach in C. Elegans [20], have revealed a complex interplay between insulin/IGF-1 signaling and ferritin expression. On the other hand, some prospective studies [16], [17] have shown a positive association between baseline levels of ferritin, i.e. the best available serum marker of body iron stores [21], and development of type 2 diabetes. On this basis, it has been postulated that iron may promote insulin resistance through its well-known pro-oxidant properties [22]. Although this causal link remains debated, it is undisputed that dysmetabolic subjects often have high serum ferritin levels, being the so-called dysmetabolic hyperferritinemia (DHF) the commonest cause of mild to moderate hyperferritinemia in clinical practice [6]. The histopathological entity known as dysmetabolic iron overload syndrome (DIOS, formerly designated as “insulin resistance-associated hepatic iron overload – IRHIO) [4] is now believed to represent the most severe clinical expression of DHF [5], where variable degrees of stainable iron coexist with classical features of NAFLD, and serum ferritin levels predicts advanced hepatic fibrosis [23].

Our view of iron overload disorders has radically changed by the discovery of hepcidin [7], which has been demonstrated to be inappropriately low in genetic hemochromatosis [8]. On the other hand, pilot studies have found high hepcidin levels in either serum [24] or urine [25] of few DIOS subjects (n = 16 to 24), suggesting a distinct pathogenesis. Supporting and extending these observations, our results establish for the first time at population level that subjects with MetS have increased serum levels of hepcidin. In subjects of both sexes hepcidin increased linearly with increasing number of the five classical MetS features, paralleling the previously described behavior of serum ferritin [14]. Of note, serum ferritin was the strongest predictor of hepcidin, while in our analyses CRP, the classical systemic marker of inflammation, was not a significant determinant of both parameters. Taken together, these data indicate that the fundamental iron regulatory feedback is preserved in MetS, i.e. that hepcidin tends to progressively increase in response to a moderate increase of iron stores, likely in the attempt to counterbalance it by limiting intestinal iron absorption. As a corollary, once simple and cheap hepcidin assays will be available in the future, the hepcidin:ferritin ratio may be proven helpful in practice for rapid distinction of DHF/DIOS from other iron overload disorders where hepcidin is inappropriately low, as mentioned above.

While our data definitively exclude hepcidin deficiency as the underlying mechanism, the key-point that remains to be addressed is the *primum movens* leading to an increase of iron stores in some dysmetabolic subjects. Aigner et al. proposed that some cytokines produced by the expanding adipose tissue (i.e. TNF-α and other “adipokines”) may down-regulate hepatic ferroportin leading to intracellular iron accumulation and compensatory stimulation of hepcidin [26]. Things are further complicated by the fact that the adipose tissue by itself may be a source of hepcidin [27]. On the other hand, some findings in women may be in agreement with these hypotheses. Indeed, we found that in women MetS was independently associated to hepcidin in multivariate models. Of note, when women with or without MetS were stratified by ferritin levels, MetS women with ferritin in the lower range had hepcidin levels significantly higher than non-MetS counterpart. Since this was particularly evident in women with ferritin levels indicating true iron deficiency (i.e. <30 µg/l) where hepcidin is generally almost completely suppressed [12], this suggests that some MetS-related factors may affect hepcidin in this subgroup. On the other hand, the influence of MetS per se on hepcidin levels appears limited when iron stores are abundant. Recent experimental studies have found that leptin, one of the main adipokines, is able to stimulate hepatic hepcidin production [28], and a positive correlation has been found between serum levels of leptin and hepcidin in obese children [29]. Our results may warrant further studies on adults in this direction, particularly focusing on differences by gender. Indeed, the reason(s) why we observed an independent influence on hepcidin only in women remain to be elucidated. Nonetheless, some clues in literature also suggest that the link between iron and dysmetabolic features may be particularly relevant in women. Sheu et al. found a relationship between ferritin and insulin resistance only in women but not in men [15]. Similarly, the largest prospective study showing ferritin as an independent predictor of future development of type 2 diabetes mellitus included only women [16].

### Chronic Hyperhepcidinemia in Metabolic Syndrome: more than Simply a Bystander?

Whatever the mechanism(s) behind, this study establishes for the first time at population level that hepcidin levels tend to be high in MetS. In view of the rapidly growing evidence for pleiotropic effects of hepcidin, this may have relevant implications for the MetS pathophysiology. First, studies in cellular models have recently demonstrated that hepcidin binding to ferroportin is able to activate Janus kinase 2/Signal Transducer and Activator 3 (Jak2/STAT3) signaling, leading in turn to an increased production of Suppressor of cytokine signaling 3 (SOCS3) [30], a central player in inducing hepatic steatosis, and MetS in mouse models [31]. Thus, hyperhepcidinemia might prime a vicious circle worsening MetS through SOCS3 induction over time. Second, high hepcidin levels may theoretically contribute to the well-known cardiovascular morbidity in MetS subjects. Indeed, three very recent experimental studies [32]–[34] have concordantly indicated that hepcidin may promote atherosclerosis, particularly by destabilizing the plaques through macrophage overactivation after erythrophagocytosis [34].

### Study Limitations

Due to its observational design, our study cannot provide any mechanistic explanation, particularly with regards to whether increased hepcidin levels are cause or consequence of insulin resistance in subjects with MetS. Similarly, the lack of data on insulin levels precluded a direct analysis of the relationship between hepcidin and estimates of insulin resistance.

### Conclusions

Our population study provides the first evidence for chronic hyperhepcidinemia as a new additional feature of MetS. The strong association between hepcidin and ferritin, as well as their parallel behavior as a function of increasing number of MetS features, suggest that hyperhepcidinemia may occur mainly in response to mild-to-moderate increase of body iron stores. Due to the recently discovered pleiotropic effects of hepcidin, our study suggests future investigations on the possible role of this hormone in worsening insulin resistance and in promoting the cardiovascular complications of MetS.

## Supporting Information

Figure S1 **Percentage of subjects with hepcidin levels in the top quartile.** (A) Males and (B) Females.
(TIF)

Figure S2 **Serum ferritin levels in the Val Borbera population according to increasing number of MetS features.** (A) whole population, (B) males and (C) females.
(TIF)

Figure S3 **Correlation between hepcidin-25 and ferritin.**
(TIF)

Figure S4 **Hepcidin levels in females according to ferritin levels and presence/absence of MetS.**
(TIF)

Figure S5 **Prevalence of MetS in females according to hepcidin levels (A), and the relative ORs for MetS, adjusted for age and ferritin (B).**
(TIF)

Table S1 **Prevalence of MetS features in the VB population.**
(DOCX)

Table S2 **Associations with hepcidin at univariate analyses.**
(DOCX)

Table S3 **Predictors of hepcidin in males and females, considering the individual MetS features as covariates.**
(DOCX)
