# Correction: Increased Serum Hepcidin Levels in Subjects with the Metabolic Syndrome: A Population Study

**DOI:** 10.1371/annotation/233a5ac3-8118-4bdc-a139-950223506864

**Published:** 2013-06-26

**Authors:** Nicola Martinelli, Michela Traglia, Natascia Campostrini, Ginevra Biino, Michela Corbella, Cinzia Sala, Fabiana Busti, Corrado Masciullo, Daniele Manna, Sara Previtali, Annalisa Castagna, Giorgio Pistis, Oliviero Olivieri, Daniela Toniolo, Clara Camaschella, Domenico Girelli

There is an error in the affiliation list for author Ginevra Biino. The following are the correct affiliations for this author:

1) Institute of Molecular Genetics, National Research Council of Italy, Pavia, Italy

2) Institute of Population Genetics, National Research Council of Italy, Sassari, Italy

