# Supplementary figures and images for: Increased Serum Hepcidin Levels in Subjects with the Metabolic Syndrome: A Population Study
Source: PLoS One. 2012 Oct 29;7(10):e48250. doi: 10.1371/journal.pone.0048250 (PMC3483177; doi:10.1371/journal.pone.0048250)

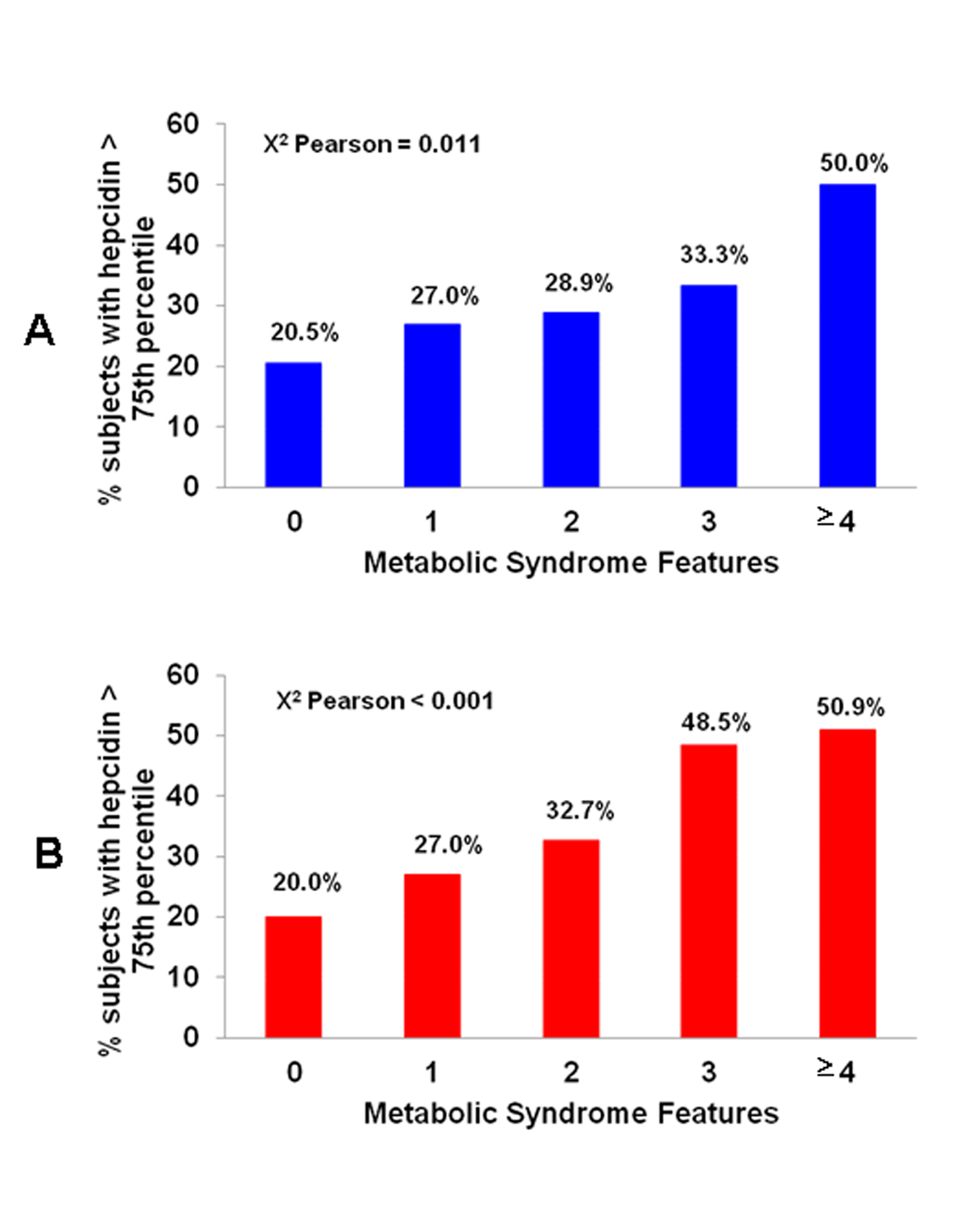

Supplement: Figure S1 — Percentage of subjects with hepcidin levels in the top quartile. (A) Males and (B) Females. (TIF) [file pone.0048250.s001.tif]

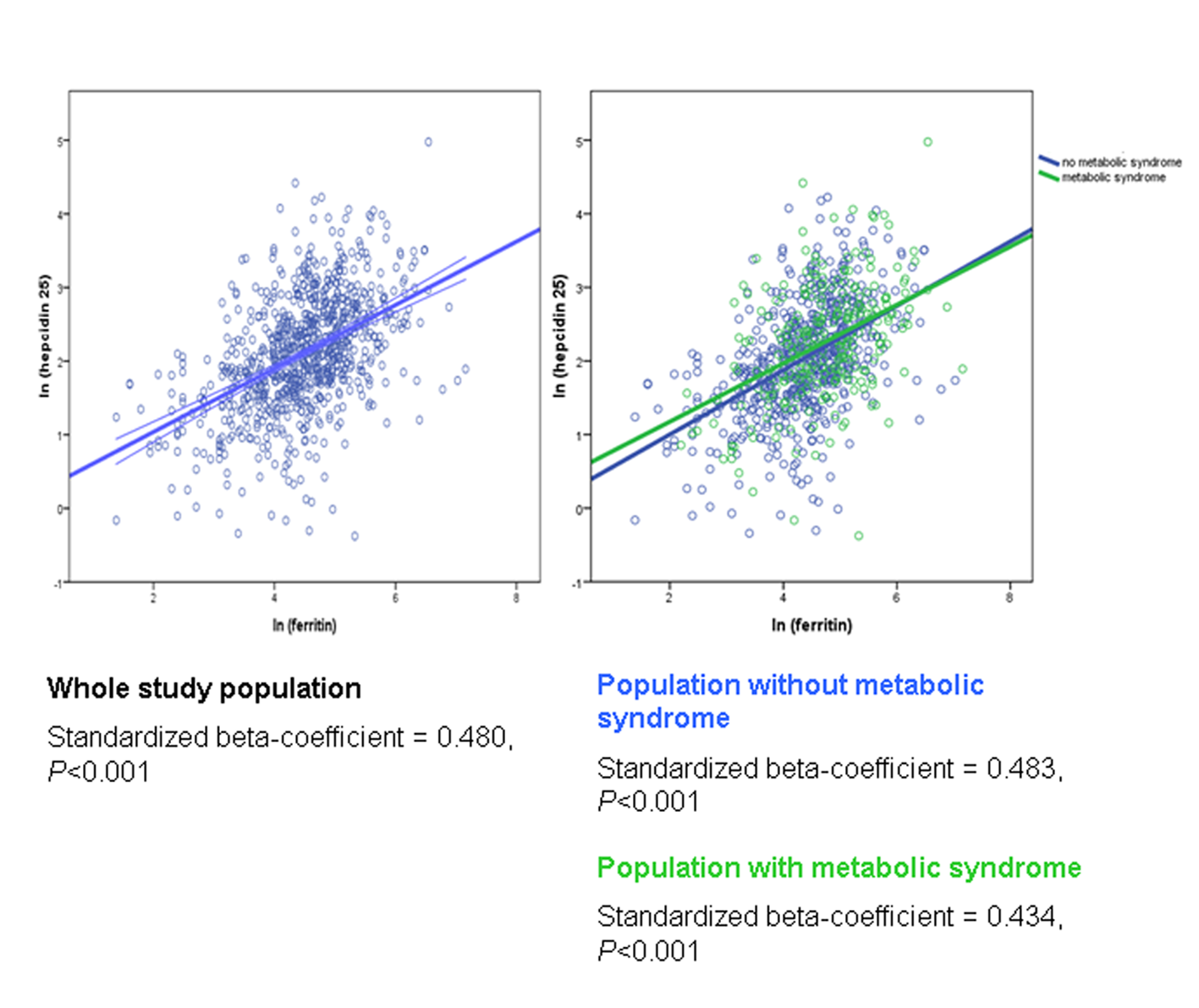

Supplement: Figure S3 — Correlation between hepcidin-25 and ferritin. (TIF) [file pone.0048250.s003.tif]

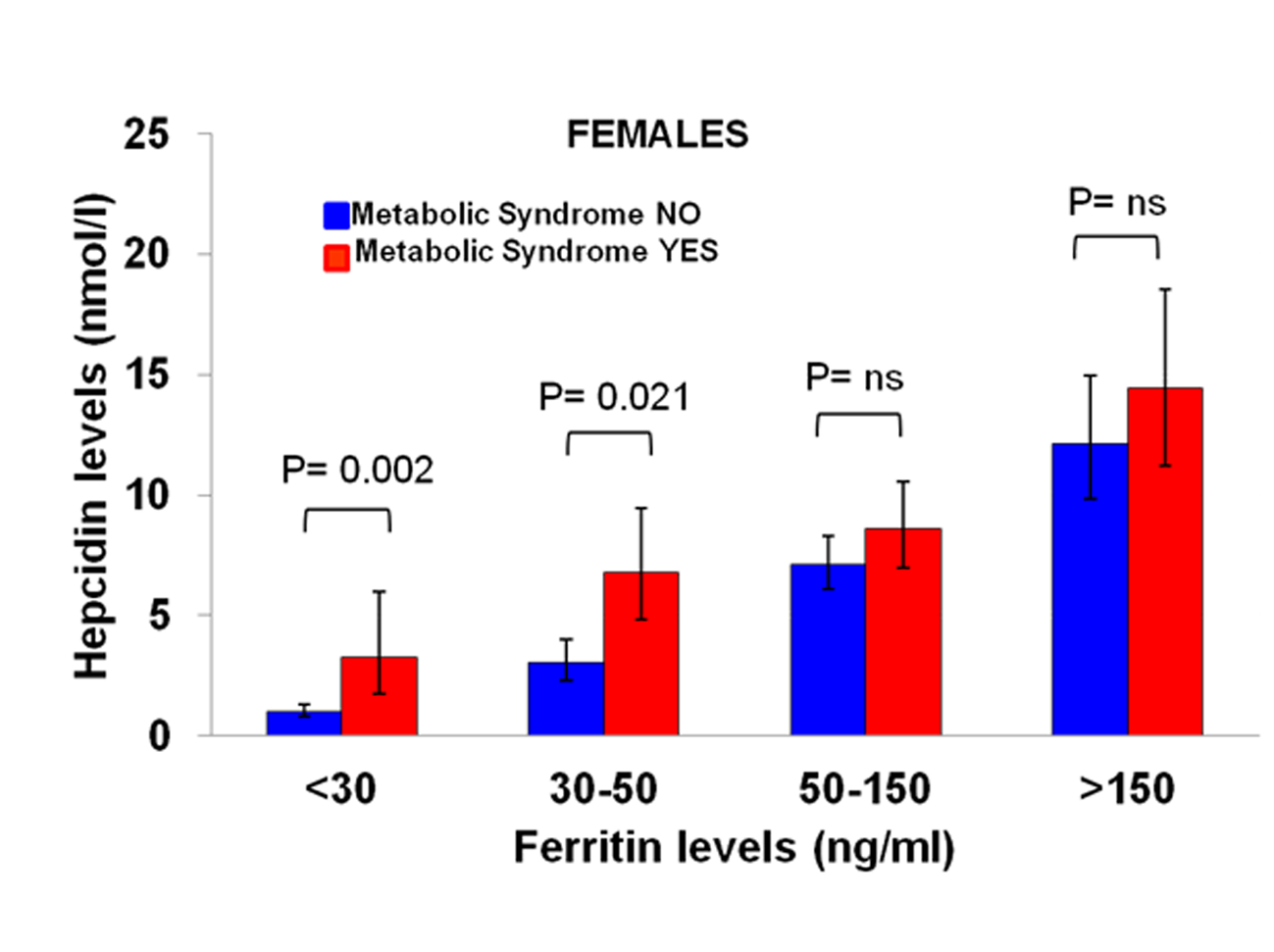

Supplement: Figure S4 — Hepcidin levels in females according to ferritin levels and presence/absence of MetS. (TIF) [file pone.0048250.s004.tif]

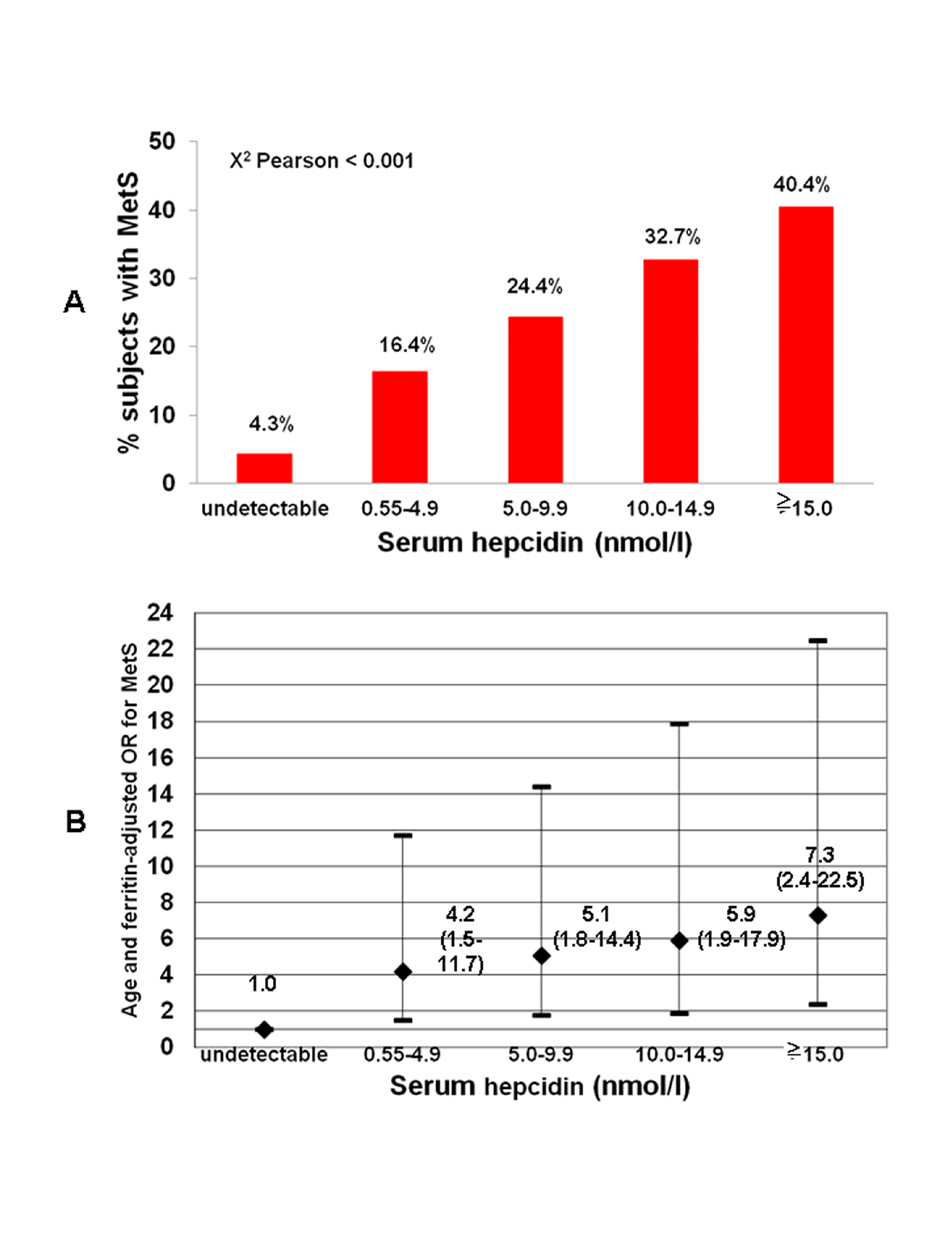

Supplement: Figure S5 — Prevalence of MetS in females according to hepcidin levels (A), and the relative ORs for MetS, adjusted for age and ferritin (B). (TIF) [file pone.0048250.s005.tif]
