# Supplementary material for: Increased Serum Hepcidin Levels in Subjects with the Metabolic Syndrome: A Population Study
Source: PLoS One. 2012 Oct 29;7(10):e48250. doi: 10.1371/journal.pone.0048250 (PMC3483177; doi:10.1371/journal.pone.0048250)
Supplement: Table S1 — Prevalence of MetS features in the VB population. (DOCX) [file pone.0048250.s006.docx]

**Table S1:** Prevalence of MetS features in the VB population

|  | **All (n = 1,391)** | **Male (n = 616)** | **Female (n = 775)** | ***P*** |
| --- | --- | --- | --- | --- |
| **Abdominal Obesity (%)** | 58.1 | 43.5 | 69.7 | < 0.001 |
| **Abnormal plasma glucose or Diabetes (%)** | 16.0 | 19.4 | 13.3 | 0.002 |
| **High Triglycerides (%)** | 15.3 | 19.0 | 12.4 | 0.001 |
| **Low HDL Cholesterol (%)** | 14.8 | 12.0 | 17.0 | 0.009 |
| **Hypertension(%)** | 68.3 | 75.8 | 62.8 | < 0.001 |
| **Metabolic Syndrome (%)** | 21.9 | 21.3 | 22.3 | 0.636 |
