# Supplementary material for: Increased Serum Hepcidin Levels in Subjects with the Metabolic Syndrome: A Population Study
Source: PLoS One. 2012 Oct 29;7(10):e48250. doi: 10.1371/journal.pone.0048250 (PMC3483177; doi:10.1371/journal.pone.0048250)
Supplement: Table S2 — Associations with hepcidin at univariate analyses. (DOCX) [file pone.0048250.s007.docx]

**Table S2:** Associations with hepcidin at univariate analyses.

|  | **Male** | | **Female** | |
| --- | --- | --- | --- | --- |
|  | **β-coefficient** | ***P*** | **β-coefficient** | ***P*** |
| **Age (years)** | -0.034 | 0.394 | 0.240 | < 0.001 |
| **Metabolic Syndrome** | 0.049 | 0.226 | 0.245 | < 0.001 |
| **S-Ferritin** | 0.559 | < 0.001 | 0.585 | < 0.001 |
