# Supplementary material for: Increased Serum Hepcidin Levels in Subjects with the Metabolic Syndrome: A Population Study
Source: PLoS One. 2012 Oct 29;7(10):e48250. doi: 10.1371/journal.pone.0048250 (PMC3483177; doi:10.1371/journal.pone.0048250)
Supplement: Table S3 — Predictors of hepcidin in males and females, considering the individual MetS features as covariates. (DOCX) [file pone.0048250.s008.docx]

**Table S3:** Predictors of hepcidin in males and females, considering the individual MetS features as covariates

|  | **Male** | | **Female** | |
| --- | --- | --- | --- | --- |
|  | **β-coefficient** | ***P*** | **β-coefficient** | ***P*** |
| **S-Ferritin** | 0.588 | < 0.001 | 0.638 | < 0.001 |
| **Abnormal Glycemia or Diabetes** | 0.010 | 0.817 | 0.080 | 0.023 |
| **Abdominal Obesity** | -0.040 | 0.348 | 0.012 | 0.737 |
| **High Triglycerides** | -0.049 | 0.256 | -0.044 | 0.231 |
| **Low HDL-C** | 0.008 | 0.854 | 0.059 | 0.101 |
| **Hypertension** | 0.010 | 0.821 | 0.001 | 0.979 |
| **Age (years)** | -0.007 | 0.875 | -0.108 | 0.013 |
